# Programmable mechanical devices through magnetically tunable bistable elements

**DOI:** 10.1073/pnas.2212489120

**Published:** 2023-04-03

**Authors:** Aniket Pal, Metin Sitti

**Affiliations:** ^a^Physical Intelligence Department, Max Planck Institute for Intelligent Systems, 70569 Stuttgart, Germany; ^b^Institute for Biomedical Engineering, ETH Zürich, 8092 Zürich, Switzerland; ^c^School of Medicine and College of Engineering, Koç University, 34450 Istanbul, Turkey

**Keywords:** mechanical metamaterials, bistability, transition waves, programmable materials, physical intelligence

## Abstract

Mechanical instabilities, especially in the form of bistable and multistable mechanisms, have recently garnered a lot of interest as a mode of improving the capabilities and increasing the functionalities of soft robots, structures, and soft mechanical systems in general. Although bistable mechanisms have shown high tunability through the variation of their material and design variables, they lack the option of modifying their attributes dynamically during operation. Here, we propose a facile approach to overcome this limitation by dispersing magnetically active microparticles throughout the structure of bistable elements and using an external magnetic field to tune their responses. We experimentally demonstrate and numerically verify the predictable and deterministic control of the response of different types of bistable elements under varying magnetic fields. Additionally, we show how this approach can be used to induce bistability in intrinsically monostable structures simply by placing them in a controlled magnetic field. Furthermore, we show the application of this strategy in precisely controlling the features (e.g., velocity and direction) of transition waves propagating in a multistable lattice created by cascading a chain of individual bistable elements. Moreover, we can implement active elements like a transistor (gate controlled by magnetic fields) or magnetically reconfigurable functional elements like binary logic gates for processing mechanical signals. This strategy serves to provide programming and tuning capabilities required to allow more extensive utilization of mechanical instabilities in soft systems with potential functions such as soft robotic locomotion, sensing and triggering elements, mechanical computation, and reconfigurable devices.

## Significance

The characteristics of bistable and multistable mechanisms are governed by their materials and design properties. Once fabricated, it has been not possible to program or tune their responses without changing their structure so far. Therefore, we demonstrate a magnetically guided strategy to precisely and predictably tune the properties of bistable elements without causing any structural or physical changes. This versatile strategy can induce bistability in monostable elements and vice-versa, modulate the energy barrier between the two stable states, and reverse the direction of switching between the two stable states. Through experiments and simulations, we demonstrate the advantages of this strategy in various applications, including programmable transition waves and reconfigurable mechanical signal-processing devices.

Mechanical instabilities are always associated with sudden large deformations. While rigid mechanisms experience irreversible plastic deformation or even failure while undergoing mechanical instabilities, soft mechanisms can utilize mechanical instabilities (without damage or failure) to increase their functionalities due to their large reversible deformability. Bistability, the property of a mechanism or structure reaching a second stable state after undergoing a mechanical instability, has seen a lot of recent interest as a method for improving behaviors and increasing functionalities in soft devices and soft robotics (1–3). Bistable mechanisms have helped soft robots overcome some of their inherent limitations and help achieve force amplification (4–6) and high-speed movements (7–10). Bistability has also allowed soft structures to attain various capabilities, such as energy absorption (11–13), deployable mechanisms (14, 15), sensing (16–18), logical computation (19–23), and metamaterial-like mechanisms (24–26). Another avenue of applications where bistable structures have been used as the building blocks are propagation of mechanical signals through a soft elastic media using transition waves (27–34).

Transition wave is the propagation of the interface between regions of two different phases as the constituent elements sequentially “transition” from one stable state to another. Also referred as the propagation of topological solitons, transition waves can be thought of as the movement of a solitary pulse in the form of the changing topology of the structural elements (35). Among the types of solitons seen in flexible metamaterials [elastic vector solitons (36, 37), rarefaction solitons (38, 39)], transition waves have gained popularity due to their high robustness arising from their input-invariant propagation characteristics (40). Recently, it has been shown that transition waves can propagate in elastic media comprising of an arrangement of flexible metamaterials. These transition waves propagate along a multiwelled energy landscape created by an array of bistable elements, each of which switch between their two stable states to propagate the transition front. Transition waves have been used to achieve unidirectional (31, 33) and bidirectional (29) propagation of signals, mechanical logic gates (27, 32), deployable structures (34), etc.

The characteristics of a transition wave, as well as that of its constituent bistable elements, however, are difficult to program or tune dynamically. The force and energy attributes of a bistable element depend on its geometric and material features (12, 20); once fabricated, they cannot be changed. A few demonstrations have used heat-responsive materials, such as shape memory alloys (41) and thermally softening polymers (42), to fabricate bistable mechanisms. However, these studies merely utilize the temperature-dependent change in material stiffness to transition between two stable states. Consequently, the setups for transition wave propagations and related functions also lack dynamic tunability. It has been previously shown that it is possible to control various aspects [e.g., velocity, direction (27, 30)], reversibility and reciprocity (43), and applications [e.g., different logical gates (27, 32) of transition waves. All this control, however, comes from physically changing or swapping various components—either the bistable elements themselves or the links connecting them—limiting their adaptability and preventing their use in situations where dynamic control is necessary. The tunability from this type of control is also limited; it is not possible to move between symmetric and asymmetric energy profiles, or choose which of the stable states is at a lower energy level. Although mechanical computing has been shown by utilizing other mechanisms as well [origami (44), vibrating beams (45), vibrating springs (46), mechanical metamaterials (47), etc.], they lack the important feature of reconfigurability.

Here, we present a facile, magnetically actuated strategy to deterministically control and program the mechanical response of tilted-beam-like bistable elements, consequently enabling the dynamic tuning of transition waves for the transmission of and computation with mechanical signals. We propose a method to utilize uniform magnetic fields to induce magnetic torques in magneto-elastomers, i.e., elastomers containing hard magnetic (ferromagnetic) microparticles with a programmed fixed magnetization direction (48–51). Previous works on changing mechanical response of a soft system with magnetic fields have focused on controlling the effective stiffness of the soft system, either by changing the viscosity of ferrofluids (52) or by physically moving a magnetic part (53, 54). In most cases, this control is binary and does not provide continuous tuning. A recent study briefly introduced the possibility of combining mechanical and magnetic loading for bistable beams, albeit without any applications (55). Our design strategy enables the capability to tune the response of intrinsically bistable beams, but also imparts bistable behavior in intrinsically monostable beams, simply by wirelessly applying an external magnetic field. The control of individual bistable elements allows us to in turn control the velocity, length, and direction of transition waves propagating along a lattice of such bistable elements without the need to change any physical components. Moreover, this strategy allows us to fabricate reconfigurable functional elements, such as a logic gate that can behave as both an AND and an OR gate, based on the direction of the applied external magnetic field.

## 1. Results

### 1.1. Design, Fabrication, and Working Principle.

**Fig. 1.**
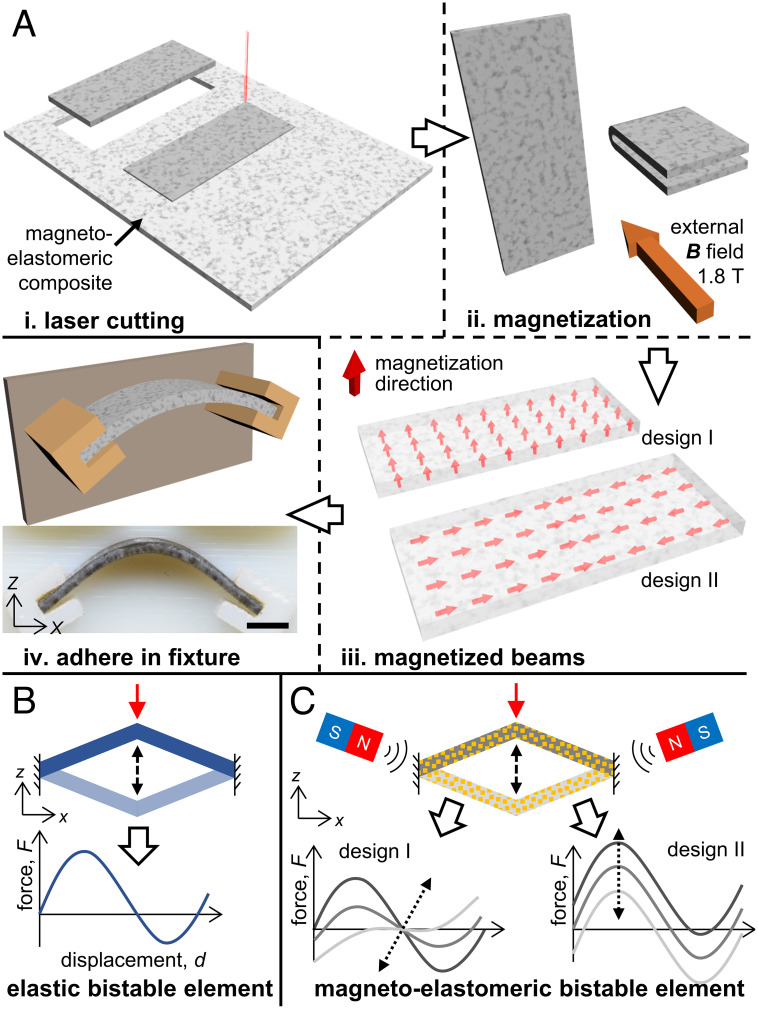
Fabrication and programmability of magnetoelastomeric bistable beams. (*A*) Schematic diagram of the fabrication process: i) a thin sheet of molded magnetoelastomeric composite (NdFeB microparticles mixed in a silicone rubber elastomer base) is laser cut into beams with a rectangular cross-section, ii) the beams are folded and oriented before being exposed to a high (1.8 T) magnetic field, iii) the magnetic domains of the NdFeB particles align with the applied field, creating the desired magnetization profile in the beam, and iv) the magnetized beams are adhered to a additively fabricated fixture using a silicone adhesive. (Scale bar: 2 mm.) (*B*) A bistable system, constituting of a mirrored, tilted elastic beam, shows the characteristic N-shaped force–displacement curve. (*C*) The force–displacement profile of a magnetoelastomeric bistable beam can be tuned in multiple ways by applying a uniform external magnetic field.

We fabricated magnetically tunable bistable mechanisms by utilizing a magnetically active elastomer composite (Fig. 1*A*). We homogeneously mixed neodymium-iron-Boron (NdFeB) hard magnetic microparticles into silicone rubber elastomer precursors, molded the magnetoelastomeric composite into thin sheets with varying thickness, and cut out rectangular beams using a solid-state laser cutter. The magnetization of the magnetoelastomeric beams was achieved by judiciously orienting and configuring them followed by placing them in a strong uniform magnetic field ( $\bar{B}$ ) of 1.8 T using a vibrating sample magnetometer (VSM). The magnetic domains of the ferromagnetic NdFeB microparticles oriented themselves with the external magnetic field and retained their orientations even after removal of the magnetization field. We programmed two types of magnetization profiles in the magnetoelastomeric beams: i) along the thickness of the beam, and ii) along the length, directed toward the center of the beam. We adhered the magnetized beams to additively fabricated fixtures to create a thin curved beam with its curvature defined by the design of the fixture. The free length (*L*) and depth (*d*) of the beam were constant throughout all experiments while the thickness (*t*) was changed to achieve varying mechanical responses.

Traditional elastic bistable elements have a fixed force–displacement (*Fd*) response, which depends on their geometry and material properties (Fig. 1*B*). Once fabricated, it is no longer possible to modify or tune the behavior and response of such elastic bistable elements. In contrast, the magnetization of the magnetoelastomeric beams allows us to tune its force and energy landscape by applying an external magnetic field and varying its magnitude and direction (Fig. 1*C*). If the magnetization profile of the magnetoelastomeric beam is along its length and a uniform magnetic field normal to the magnetization direction is applied (*z* direction), the *Fd*-curve is translated linearly along the force axis. The direction and magnitude of the translation depends on the direction and magnitude of the applied magnetic field. Similarly, if the magnetization is along the thickness of the beam, the external magnetic field scales the curvature of the *Fd*-curve; i.e., it varies the energy stored and released by the bistable beam while transitioning from one stable state to the other (Fig. 1*C*).

### 1.2. Inducing Bistability in Inherently Nonbistable Systems.

**Fig. 2.**
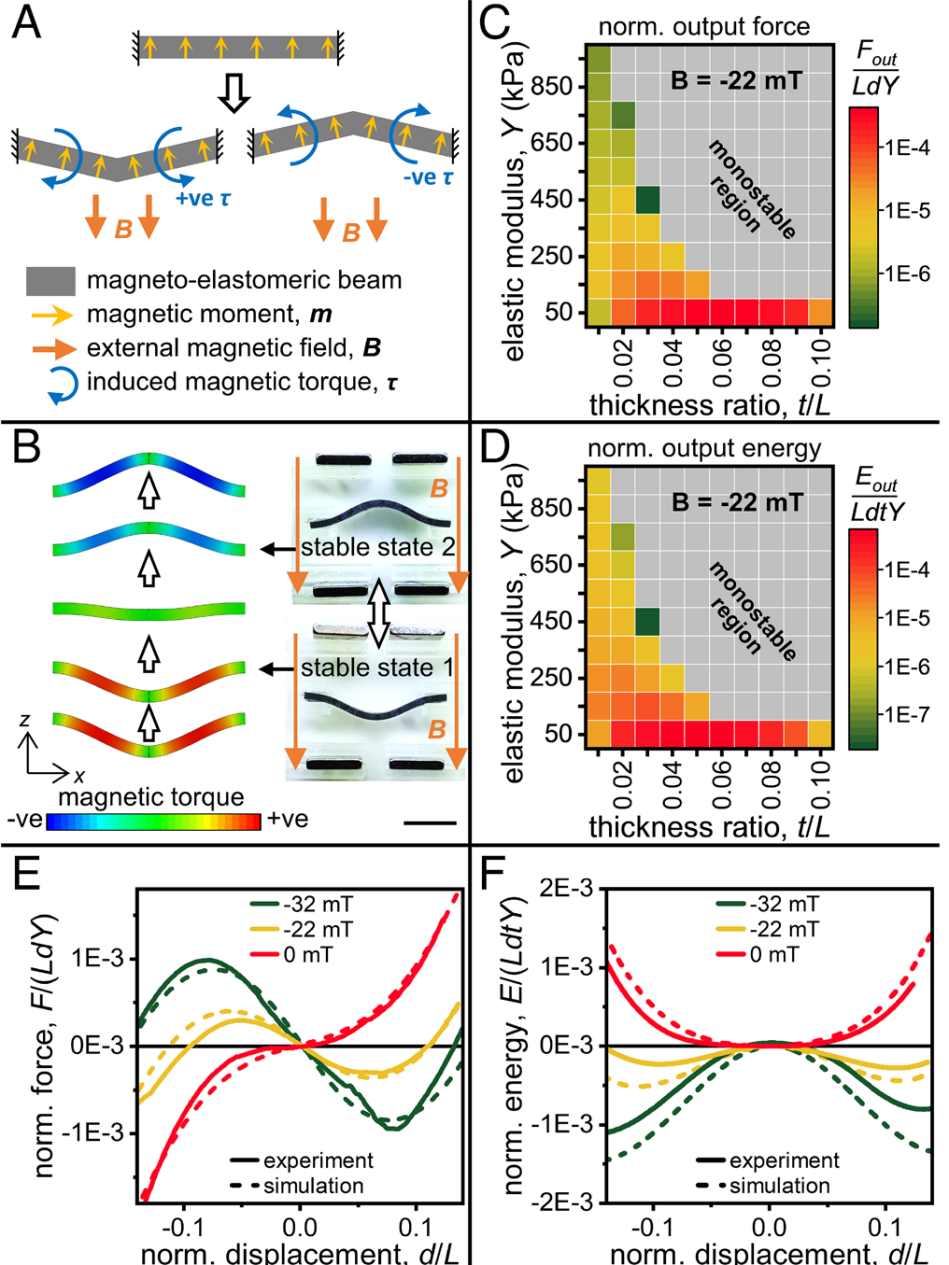
Induction of tunable bistability in straight magnetoelastomeric beams. (*A*) Schematic showing the magnetization profile of the magnetoelastomeric beam, direction of the applied magnetic field, and the resultant magnetic torque on the beam. The torques always reinforce the bending of the beams. (*B*) Finite-element simulations showing the evolution of the torques generated along the beam (under to a magnetic field of *B_z_* = −22 mT) as it is displaced vertically. The simulations were performed for half the beam using symmetry conditions and mirrored to show the entire beam. *Insets* show the two stable states of the beam. (Scale bar denotes 5 mm.) (*C* and *D*) Effect of *Y* and *t*/*L* on the normalized maximum output force and normalized energy released by the system while transitioning to the second stable state (*B_z_* = −22 mT). The gray regions indicate combinations where bistability was not achieved. (*E* and *F*) Normalized force and energy profiles, from experiments and FE simulations, of a magnetoelastomeric beam (elastic modulus, *Y* = 50 kPa and thickness-to-length ratio, *t*/*L* = 0.04) under varying magnetic fields.

A straight beam, fixed at both ends, is inherently monostable. When the center of the beam is displaced (normal to its length), it leads to an increase of elastic energy due to stretching of the beam. Here, we introduce a strategy to convert such a beam into a bistable system. We magnetize a magnetoelastomeric beam along its thickness and place it in a position with no initial bend or curvature. When an external uniform magnetic field is applied in a direction opposite to the magnetization direction, the beam exhibits bistable behavior under further mechanical loading; i.e., after displacement to either side, the beam can maintain its position without any external mechanical force. The induced magnetic torque (***τ***) generated can be expressed as follows:

[1] $$\tau \;=\;\bar{m}\;\times \;\bar{B}$$

where $\bar{m}$ is the magnetic moment and $\bar{B}$ is the applied uniform magnetic field (Fig. 2*A*). Considering the defined coordinate system, the torque can also be expressed as *m_x_B*, where *m_x_* is the component of magnetization in the *x* direction. In its initial (undeformed) state, *m_x_* = 0, and therefore there is no torque. A small deflection of the beam (in either direction), however, creates a nonzero *m_x_* and induces a magnetic torque which reinforces the bending. This causes *m_x_* to increase, leading to the continuous increase of displacement and torques. The beam reaches a stable equilibrium point when the stress generated due to the stretching of the beam balances the stresses generated due to the induced magnetic torque. Two such stable equilibrium points exist on either side of the initial beam position, creating a bistable system (Fig. 2*B* and Movie S7). It should be noted that the zero-displacement (undeformed) state of the beam is an unstable equilibrium point.

We use finite-element simulations to study the response of beams fabricated with varying material properties (elastic modulus, *Y*) and geometries (thickness-to-length ratio, *t*/*L*) (Fig. 2 *C* and *D*). We utilize the parameters of normalized output forces (*F_out_*/*LdY*) and energies (*E_out_*/*LdtY*) as a measure of the bistability beam (*SI Appendix*, Fig. S1). Softer materials generate larger forces *F_out_* and energies *E_out_*, as decreasing the stiffness reduces the stresses generated due to stretching and allows larger deformation before the torque and deformation stress negate each other. An optimal *t*/*L* (0.06) can be found: A small thickness reduces the volume-dependent magnetic moment, and in turn the torque, while a large thickness increases bending stiffness, reducing the torque-based deformation. Fig. 2 *E* and *F* shows the force and energy characteristics of a beam magnetized in the positive *z* direction and fabricated with a stiffness, *Y* = 50 kPa, and thickness-to-length ratio, *t*/*L* = 0.06, under varying magnetic fields. At ***B*** = 0, the undeformed state is the only stable state and the energy increases with displacements in either direction; applying a negative magnetic field induces bistability in the same beam. The numerical results show good accordance with the experimental results. The magnitude of the magnetic field affects the induced magnetic torque; increasing the magnitude of $\bar{B}$ moves the two stable states further apart as well as increases the energy barrier between them. By controlling the external magnetic field strength, we can not only generate bistable systems from an inherently non-bistable element, but also tune the degree of its bistability in a deterministic way.

### 1.3. Tuning the Asymmetry of Stable States in a Bistable System.

We can further improve the energy profile of the previously described magnetoelastomeric bistable systems and introduce asymmetry between the two stable states. Such an asymmetry opens up the way for applications that require directional state transition, such as the propagation of topological solitons or transition waves. We use a linear array of inherently asymmetric bistable elements to create a system that supports the propagation of transition waves; we then locally apply magnetic fields to individual elements to tune their responses and program the behavior of the transition wave. We modify the magnetization profile to be along the length and directed toward the center of the beam while maintaining the direction of the external magnetic field (along the thickness of the beam). We also modify the initial state of the beam by introducing a curvature, quantified by the angle (*θ*) made by the end of the beam. These initially curved beams have the potential to be intrinsically bistable, based on their geometry and stiffness. We used the relatively stiffer polydimethylsiloxane (PDMS) elastomer (stiffer compared to Ecoflex 00-30 silicone rubber) as the increased stiffness led to higher absolute output forces and energies, which can be used for useful work. Note that this strategy is compatible with softer materials as well and the normalized output force and energy would remain the same.

**Fig. 3.**
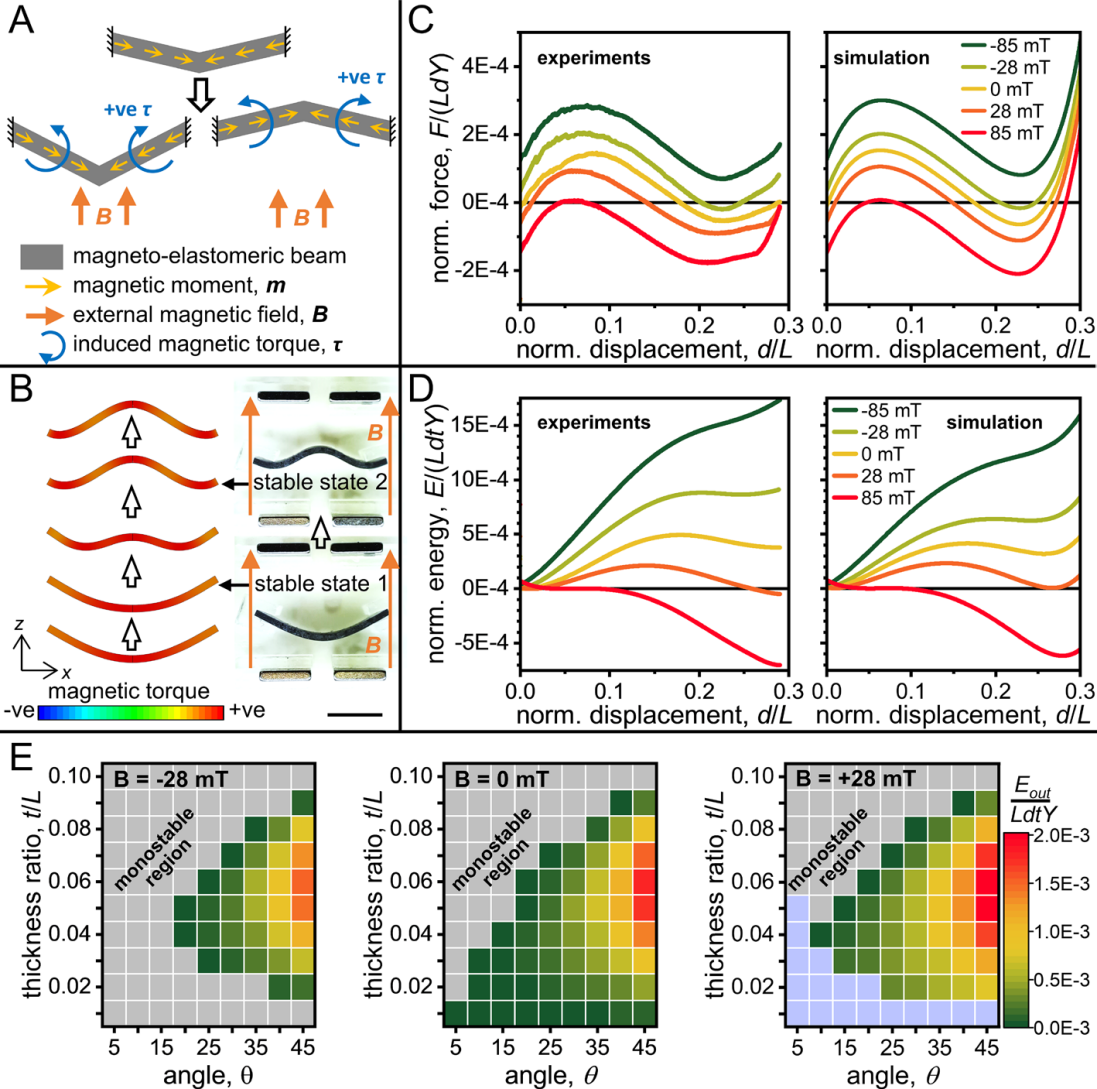
Tuning the characteristics of bistable magnetoelastomeric beams. (*A*) Schematic showing the magnetization profile of the magnetoelastomeric beam, direction of the applied magnetic field, and the resultant magnetic torques on the beam. The torque directions are not dependent on the bending direction of the beam. (*B*) FE simulations showing the evolution of the torques generated along the beam (under to a magnetic field of *B_z_* = 28 mT) as it is displaced vertically. The simulations were performed for half the beam using symmetry conditions and mirrored to show the entire beam. *Insets* show the two stable states of the beam. (Scale bar denotes 5 mm.) (*C* and *D*) Normalized force and energy profiles, from experiments and FE simulations, of a magnetoelastomeric beam (initial angle, *θ =* 20° and thickness-to-length ratio, *t*/*L* = 0.04) under varying magnetic fields. (*E*) Effect of *θ* and *t*/*L* on the normalized maximum output force and normalized energy released by the system while transitioning to the second stable state (*B_z_* = 0, −28, and +28 mT). The gray regions indicate combinations where bistability was not achieved. The blue regions indicate where the *s*_1_ became the only stable state.

When a uniform external magnetic field is applied, the induced magnetic torque maintains its sign, irrespective of the bending direction of the beam (Fig. 3*A*). This constant torque direction works to linearly translate the *Fd*-curve along the force axis. The direction of translation depends on the direction of the applied magnetic field. Fig. 3 *C* and *D* shows the response of a beam with *t*/*L* = 0.04, *θ =* 20°, under varying magnetic fields. The beam is inherently bistable, with its undeformed (stable) state (*s_0_*) being more energetically favorable than the deformed stable state (*s*_1_), *Es*_0_ < *Es*_1_. When a small positive magnetic field (*B* = 28 mT) is applied, torques bending the beam upward are induced, and the two stable states (*Es*_0_ = *Es*_1_) become equally energetically favorable. If the magnetic field is increased (*B* = 85 mT), the deformed state (*s*_1_) actually becomes more energetically favorable than the undeformed one (*Es*_0_ > *Es*_1_). Conversely, if the magnetic field is applied in the opposite direction (*B* = −85 mT), the system no longer remains bistable; the undeformed state remains the only stable state. Similar trends under varying magnetic fields are also seen from experiments and simulations for magnetoelastomeric beams with different *t*/*L* or *θ* values (*SI Appendix*, Fig. S2).

Fig. 3*E* and *SI Appendix*, Fig. S3 show the results of parametric study of beams with varying geometries under zero, positive, and negative uniform magnetic fields. Negative fields move the *Fd*-curves upward; some beams which were intrinsically bistable become monostable. Positive fields move the *Fd*-curves downward; some intrinsically monostable systems become bistable. Some inherently bistable systems, however, also become monostable; this happens when the deformed stable state (*s*_1_) becomes the only stable state (indicated by the blue regions in the plot on the right in Fig. 3*E*). Compared to the previous section, the current configuration adds the advantage of being able to use inherently bistable beams and then further tune their behavior as necessary. This helps to simplify large systems with arrays of bistable elements (as in the following sections), where a local magnetic field can be applied to modify responses locally; as opposed to each element requiring a magnetic field simply to become bistable.

The ends of the beams here are fixed, aligned with the initial curvature of the beam (Fig. 3 *A* and *B*). This alignment, without any magnetic fields, leads to the undeformed stable state to be automatically at a lower energy level than the deformed stable state (*Es*_0_ <*Es*_1_). If the ends of the beam are fixed without any initial inclination, then the deformation becomes symmetrical on both sides; consequently, the two stable states also remain at equal energy levels (*Es*_0_ = *Es*_1_) (*SI Appendix*, Fig. S4*A*). The *Fd*-curves of bistable beams with straight boundary conditions can also be tuned using magnetic fields. *SI Appendix*, Fig. S4 *B* and *C* shows the force and energy profiles of a beam with *t*/*L* = 0.06, *θ =* 15, where, initially, both its stable states are at the same energy level, but under different directions of magnetic fields, any one of the stable states can be made to be at a lower energy than the other. *SI Appendix*, Fig. S4*D* shows the results of a parametric study for this configuration.

### 1.4. Programming the Propagation of Transition Waves: Length, Direction, and Velocity.

Bistable systems lend themselves particularly well as a lattice element for transition wave propagation. Consecutively linking multiple bistable elements in a lattice creates an energy landscape with multiple minima, with each minimum corresponding to a stable configuration. As a transition wave propagates through the lattice, it shifts each individual element from one stable state to another more energetically favorable one. The energy characteristics of the two stable states of the individual bistable element dictate the properties (e.g., directionality, velocity) of the transition wave.

We created a one-dimensional (1D) periodic lattice of our magnetoelastomeric bistable beams, connected by linear elastic links. These inherently bistable beams were magnetized along their length, and a uniform magnetic field was used to change their properties individually or collectively as necessary. We introduce the normalized displacement for each beam ( ${\bar{z}}_{k}$ for beam *k*), with respect to their two stable states (*s*_0_ and *s*_1_), to characterize the propagation of the transition wave.

**Fig. 4.**
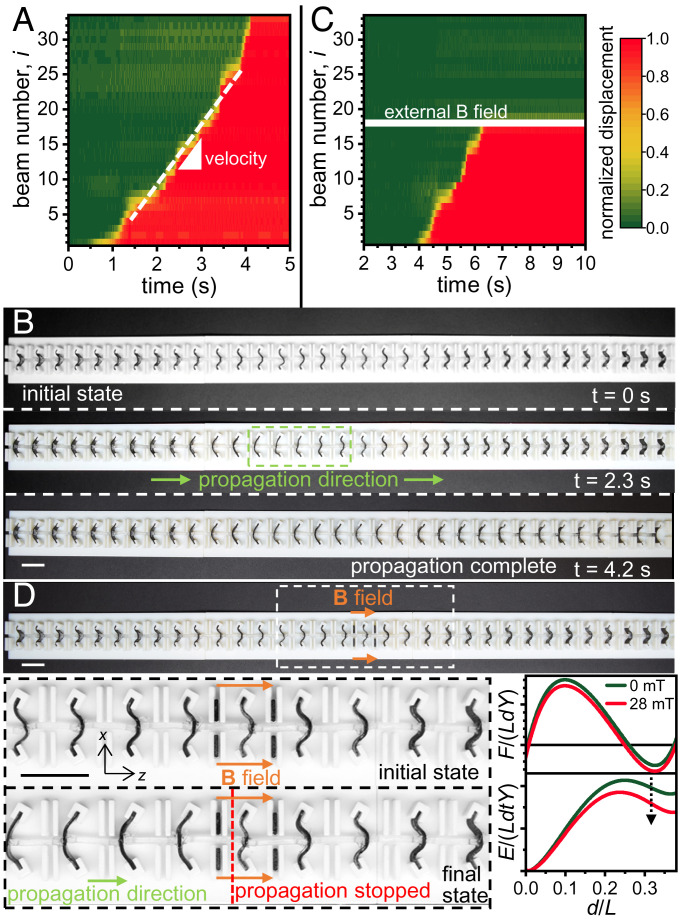
Transition waves in a lattice of bistable beams and magnetic control of a transistor-like system for mechanical signals. (*A*) Time evolution of the normalized displacements for each bistable beam ( $z_{k}$ for beam *k*) as a transition wave propagates along a lattice of such beams. ${\bar{z}}_{k}=0,1$ indicates the two stable states. The slope of the boundary between the two stable regions indicates the velocity of the transition wave. (*B*) Snapshots from Movie S1 showing the lattice of bistable beams at the beginning of, during, and at the completion of the propagation of the transition wave. (*C*) Time evolution of the normalized distances of a transistor-like device, where an external magnetic field (here applied to beam #18) can be used similar to a gate to control the propagation of the transition wave. (*D*) A lattice of bistable beams with an external magnetic field applied at the middle. *Insets* are snapshots from Movie S2, showing the discontinuance of the transition wave propagation at the location of the applied magnetic field. The magnetic field increases the energy barrier between the two stable states. (Scale bar: 10 mm.)

[2] $${\bar{z}}_{k}=\left|\frac{z_{s0}-z_{k}}{z_{s0}-z_{s1}}\right|,$$

where *z_k_* is the coordinate of the *k*th bistable beam, and *z*_*s*0_, *z*_*s*1_ are the coordinates of two stable states. First, we observed the propagation of a transition wave in a lattice of bistable beams (*t*/*L* = 0.06, *θ* = 30°) without any external magnetic field and extracted the time-displacement data for each beam. All the bistable elements are placed in their higher energy stable state and a mechanical perturbation is applied to one end (left) of the lattice (large enough to switch the first bistable element), causing a transition wave to propagate outward. The time evolution of the normalized displacement ( $z_{k}$ ) data can be used to calculate the speed of the transition wave; the boundary between the two stable regions indicates beams in transition, and the (constant) slope of the boundary indicates the speed of the wave (8 units s^−1^) (Fig. 4*A*). Fig. 4*B* shows different stages of the propagation of the transition wave: before the start of, during, and at the completion of propagation (Movie S1).

We then applied a local magnetic field along the positive *z* direction (*B_z_* = 28 mT), using small permanent magnets, to one bistable element (#18) in the middle of the lattice, and lowered the energy of the deformed stable state (*s*_1_). When a perturbation was applied to the lattice, a transition wave was generated as before, but its propagation stopped at the element with the modified energy levels (Fig. 4*C*). The increased energy barrier between the two stable states stopped the switching of the bistable element and prevented the continuation of the transition wave (Fig. 4*D* and Movie S2). This ability to wirelessly control the propagation length of the transition wave, without the need to physically modify the lattice elements, is critical for controllable mechanical signal transmission through an elastic medium.

**Fig. 5.**
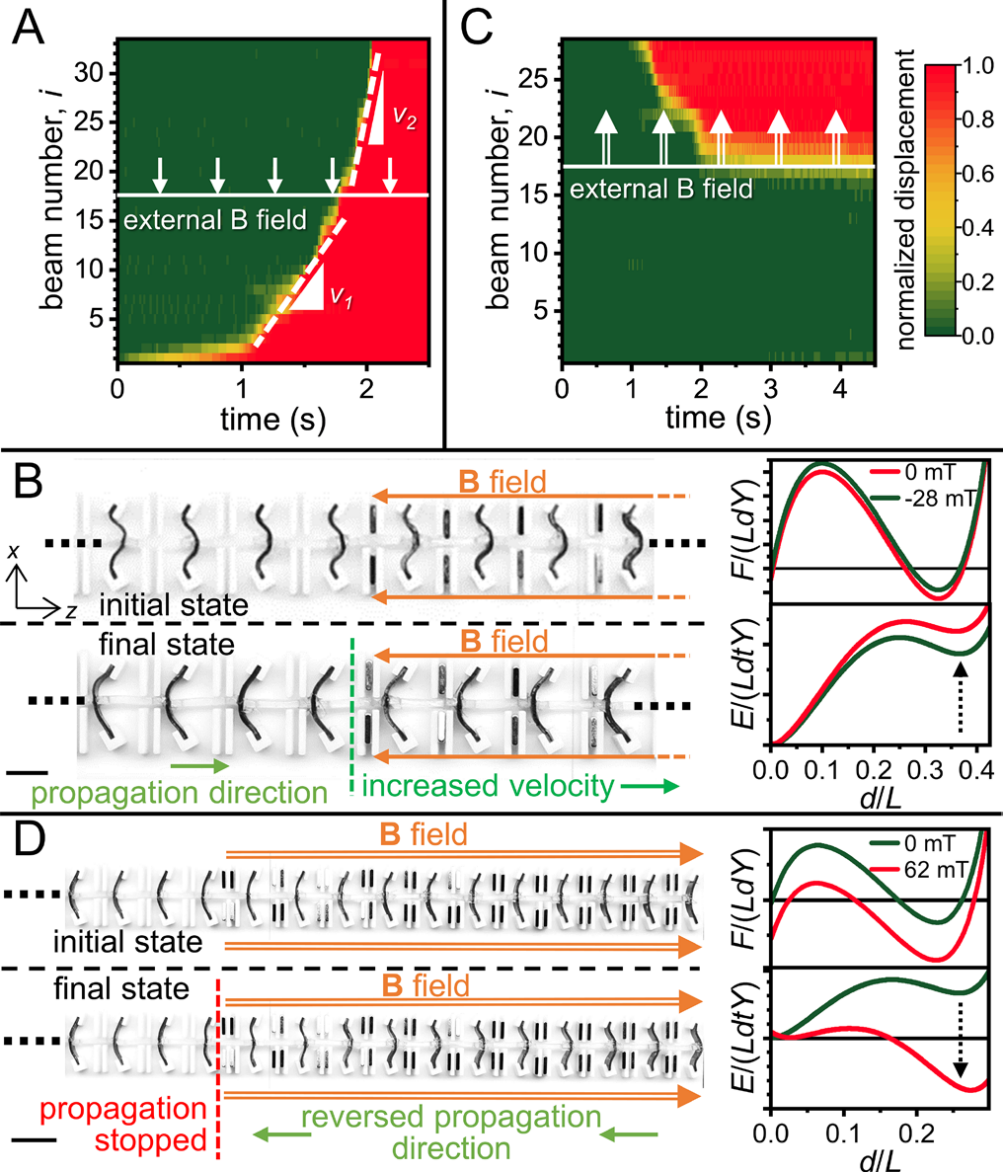
Magnetic control of the velocity and direction of the transition waves. (*A*) Plot of normalized displacements showing two different velocities at different sections of the lattice. (*B*) Snapshots from Movie S3 showing the application of a uniform magnetic field along -​*z* direction over one half of the lattice, leading to an increase in the transition wave velocity. Graphs show the reduction of energy barrier between the two stable states due to the magnetic field. (*C*) Plot of normalized displacements showing the reversal of transition wave propagation direction. (*D*) Snapshots from Movie S4 showing that the application of a magnetic field along +*z* direction causes the transition wave from right to left. The graphs show how the magnetic field makes the second stable state to be at a lower energy level than the first. (Scale bar: 5 mm.)

The high tunability of each individual bistable element also allows us to control and modify other characteristics of the transition wave. If we reverse the direction of the magnetic field (negative *z* direction), the energy differential between the two stable states increases and the energy barrier decreases. This works to increase the speed of the transition wave. Fig. 5 *A* and *B* shows a 1D lattice made with bistable beams (*t*/*L* = 0.06, *θ* = 30°) with a negative magnetic field (*B_z_* = −28 mT) applied over half of the length of the lattice (Movie S3). The slope of the transition region of the normalized displacement graph shows a sharp increase midway, indicating the increase of speed due to the external magnetic field. It should be noted that even though the absolute energy level increase of the second stable state is not very high, the relative reduction of the energy barrier is significantly high, causing the speed increase. We can also achieve the complete reversal of the transition wave propagation direction by applying sufficiently high magnetic fields. Fig. 5 *C* and *D* shows a 1D lattice made with bistable beams (*t*/*L* = 0.04, *θ* = 20°) set in position *s_0_*, with a high positive magnetic field (*B_z_* = 62 mT) applied over one end of the lattice. This setup allows a transition wave to propagate along the -*z* direction, stopping at the end of the magnetic field region (Movie S4). Under the influence of the magnetic field, *s_1_* becomes the lower energy stable state, allowing the beams to transition from *s*_0_ to *s*_1_ and the wave to reverse its propagation direction.

### 1.5. Programmable Logic Gates.

**Fig. 6.**
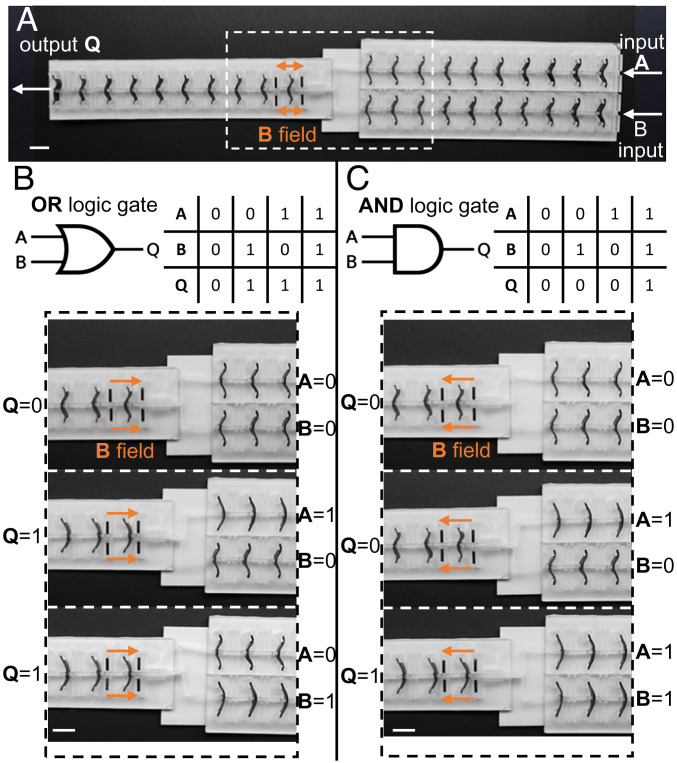
Functional devices that can be reconfigured magnetically. (*A*) A T-shaped (bifurcated) lattice of bistable elements with two inputs (**A** and **B**) and one output (**Q**). (*B* and *C*) Snapshots from Movies S5 and S6, showing how the magnetic field direction changes the functionality of the gate between a logical **OR** gate (*B*) and a logical **AND** gate (*C*). *Insets* show multiple input combinations and their corresponding outputs for both the gates. (Scale bar: 10 mm.)

We use the principle of using external magnetic fields to tune bistable beams to develop programmable functional devices (e.g., mechanical logic gates) for binary logical operations with mechanical signals. We created a binary logic gate with two inputs (**A** and **B**) and one output (**Q**), connected by a T-shaped flexible link (Fig. 6*A*). The high energy state (*s*_1_) can be defined as the logical state **0**, and the low energy state (*s*_0_) as **1**. A magnetic field applied to the first element of the output chain can be used to dynamically reconfigure the operation of the binary logic gate between an **AND** gate and an **OR** gate. If a positive magnetic field is applied, the energy differential between *s*_1_ and *s*_0_ decreases, allowing only one input to cause the propagation of the output chain (**OR** gate) (Fig. 6*B* and Movie S5). Conversely, if a negative magnetic field is applied, the energy differential increases, requiring the activation of both inputs for the output chain propagation, realizing an **AND** gate (Fig. 6*C* and Movie S6). This strategy allows us to program the same physical system to act as different logical gates under different magnetic fields; i.e., there is no need to change or use different beams, links, etc., merely changing the applied magnetic field can dynamically realize different logical components.

## 2. Discussion

Here, we introduced a strategy of using magnetic fields to wirelessly and dynamically tune the mechanical responses of bistable systems, providing the capability to program and manipulate the propagation of mechanical signals through elastic media, as well as allowing on-demand reconfiguration of functional devices for binary logic operations. We demonstrate that this strategy is easy to implement, simple to fabricate, and possible to extend using a modular approach. We establish experimentally and verify numerically the following contributions in this work: i) the realization of magnetic field-induced bistability in inherently monostable structures; ii) magnetically controlled tuning of the output characteristics of inherently bistable beams, including choosing the energetically favorable stable state, and changing to a monostable configuration; iii) complete control of the attributes (e.g., velocity, length, direction) of transition waves as well as achieving gate-controlled transistor-like functionalities through the manipulation of wirelessly applied magnetic fields; iv) magnetically reconfigurable logical elements: a single physical system which can function as both a logical **AND** and a logical **OR** gate, controlled by the direction of an external magnetic field.

This work has two limitations at the present level of development. First, the magnetic control and its analysis is based on the torques generated from uniform magnetic fields. Limiting ourselves to uniform fields allows us to easily realize symmetry conditions and simplify the implementation of the numerical model. The magnetic forces arising out of diverging magnetic fields, however, can also be utilized to actuate the bistable elements, allowing their use as sensory or triggering elements in completely untethered soft robots. Second, the uniform magnetic fields are generated with permanent magnets, placed on both sides of the bistable elements. The use of permanent magnets allows us to easily apply a local magnetic field as well as modify its magnitude and direction, while the parallel configuration of the permanent magnets ensures uniformity of the magnetic fields. It also allows us to circumvent the need of high-power consuming coils and their associated overheating issues. If necessary, the Helmholtz coils can be used to generate similar uniform magnetic fields and streamline its regulation through electronic control.

The strategy to reconfigure devices for multiple purposes is well suited for additive manufacturing techniques like stereolithography (mm to cm scale) and two-photon polymerization (µm scale). Scaling down to micron scales would also lead to an increase in the density of computational elements, allowing for more complex circuits and higher computational capabilities. Similarly, this approach can also be scaled up for applications such as haptics and human–computer interactions. The nondimensionalized parameters used in this study allow the results to be used at any size scale and the transformations are scale independent, while the gravitational effects and creation of uniform fields in large workspaces need to be taken care of carefully at much larger length scales. An increase in computational efficiency can also be achieved by incorporating higher dimensional arrays of bistable elements to support control and propagation of mechanical signals’ three-dimensional (3D) space. Various other reconfigurable logic elements can also be easily achieved by extending the principles demonstrated in this work, leading to the development of higher-order reconfigurable logic circuits, such as adders and registers.

Bistable and multistable systems have recently become indispensable in soft robots and mechanisms, increasing their capabilities and functionalities. In this work, we demonstrate a facile strategy which provides exhaustive, untethered tunability of bistable systems with wirelessly applied external magnetic fields, enabling the control of transition waves and the realization of programmable and reconfigurable functional elements such as transistors and logic gates. The programmable logical processing shown here can be used to introduce more sophisticated physical intelligence (56) and autonomy in entirely soft robots, without the need of, or even surpassing the capabilities of rigid silicon-based electronics. We envision that this easily implementable strategy will pave the way for soft robots to access the advantages of tunable, on-demand bistability and achieve previously unavailable functionalities in motion, control, sensing, and computation.

## 3. Materials and Methods

### 3.1. Characterization.

#### 3.1.1. Mechanical characterization.

Tensile tests of dogbone-shaped samples of the magnetoelastomeric materials were performed according to ASTM D412-C specifications to determine the elastic modulus of the materials. A universal testing machine (Instron 5942), with 10 N and 50 N load cells, was used to perform the experiments at a crosshead loading rate of 5 mm min^−1^. The materials were found to be linearly elastic with an elastic modulus of 44 kPa and 4.9 MPa for the Ecoflex and PDMS-based elastomeric composites, respectively (*SI Appendix*, Fig. S5).

#### 3.1.2. Magnetic characterization.

We determined the magnetization strength of our materials using a VSM (Micro Sense Ez7). We cyclically applied an externally magnetic field (***H***) while measuring the magnetization (***M***) of the sample of known size and weight. We obtained the magnetic hysteresis curve from this procedure and determined the residual magnetization (*M_r_*) as 104.91 A mm^−1^ (*SI Appendix*, Fig. S6) (57).

#### 3.1.3. Characterization of permanent magnet setups.

We used FE simulations to find the magnetic fields generated by the permanent magnets used in the experiments and verified them experimentally using a handheld Gaussmeter (*SI Appendix*, Fig. S7). We used the AC/DC module of COMSOL Multiphysics^®^, version 6.0, to perform the simulations. The properties of the permanent magnets were defined by their residual magnetism, as obtained from their data sheets. We obtained primarily uniform magnetic fields with minimal divergence inside our area of interest by placing the permanent magnets in a parallel configuration.

### 3.2. Fabrication.

#### 3.2.1. Fabrication of magnetoelastomeric composites.

All magnetoelastomeric composites were fabricated by mixing and degassing magnetic microparticles in uncured elastomers in a planetary mixer and for 90 s. The magnetoelastomeric composites were blade casted on a poly(methyl methacrylate) substrate, and a heat-resistant, polyester backing tape was used as the spacer. The blade-casted sheets were cured in a 90 °C oven for 4 h followed by laser cutting to obtain rectangular beams.

#### 3.2.2. Fabrication of the transition wave setup.

The frames to hold the magnetoelastomeric beams were 3D printed with a rigid material. The linear spring elements (links) are 11 mm long and were fabricated by curing a composite of PDMS and Ecoflex 00-30 (1:1) in 3D-printed molds coated by mold release (*SI Appendix*, Fig. S8). The linear spring elements and the magnetoelastomeric beams were joined using a silicone adhesive and a custom 3D-printed fixture was used to help align consecutive beams and links. The ends of the bistable beams fit snugly in the frame (maximum gap 200 µm), minimizing free movement or rotation of the ends.

### 3.3. Mechanical Response Measurement.

A custom-made tensile testing setup with a 1 N load cell and high-precision moving stages were used to characterize the load-displacement responses of the magnetoelastomeric beams. The beams were fixed in custom 3D-printed frames with different angles which were then placed on the *xy*-stage. The *z*-stage was moved at a speed of 0.1 mm s^−1^ and the load-displacement data were recorded at a frequency of 1,000 Hz.

### 3.4. Finite-Element Analysis.

Finite-element analysis was performed to model the tunable bistable behavior of the magnetoelastomeric beams. All finite-element simulations were performed with a commercial finite-element solver (Abaqus/Standard, version 2020, Simulia, Dassault Systèmes) using two-dimensional shell elements. Dynamic/implicit analyses were performed to capture the instabilities of the responses of the magnetoelastomeric beams. The magnetic torques on the beams were calculated with a custom user subroutine (UAMP), which was used to update the magnitude and direction of 3D torque values at each node, based on their 3D orientations at each increment.

## Supplementary Material

Appendix 01 (PDF)

Movie S1. Propagation of a transition wave in a lattice of bistable elements

Movie S2. Transistor like control of transition wave propagation

Movie S3. Magnetic control of velocity of transition waves

Movie S4. magnetic control of the direction of propagation of transition waves

Movie S5. Magnetic control of the functionality of a binary logical element: **OR** gate

Movie S6. Magnetic control of the functionality of a binary logical element: **AND** gate

Movie S7. Introducing bistability in an inherently monostable beam

## Data Availability

All study data are included in the article and/or *SI Appendix*.

## References

[1] A. Pal, V. Restrepo, D. Goswami, R. V. Martinez, Exploiting mechanical instabilities in soft robotics: Control, sensing, and actuation. Adv. Mater. **33**, 2006939 (2021). 10.1002/adma.202006939 33792085

[2] M. Li, A. Pal, A. Aghakhani, A. Pena-Francesch, M. Sitti, Soft actuators for real-world applications. Nat. Rev. Mater. **7**, 235–249 (2022). 35474944 10.1038/s41578-021-00389-7 PMC7612659

[3] H. Yasuda , Mechanical computing. Nature **598**, 39–48 (2021). 34616053 10.1038/s41586-021-03623-y

[4] A. Pal, D. Goswami, R. V. Martinez, Elastic energy storage enables rapid and programmable actuation in soft machines. Adv. Funct. Mater. **47907**, 1906603 (2019).

[5] T. Chen, O. R. Bilal, K. Shea, C. Daraio, Harnessing bistability for directional propulsion of soft, untethered robots. Proc. Natl. Acad. Sci. U.S.A. **115**, 5698–5702 (2018). 29765000 10.1073/pnas.1800386115 PMC5984517

[6] Y. Tang , Leveraging elastic instabilities for amplified performance: Spine-inspired high-speed and high-force soft robots. Sci. Adv. **6**, eaaz6912 (2020). 32494714 10.1126/sciadv.aaz6912 PMC7209986

[7] R. V. Martinez , Robotic tentacles with three-dimensional mobility based on flexible elastomers. Adv. Mater. **25**, 205–212 (2013). 22961655 10.1002/adma.201203002

[8] R. Baumgartner , A lesson from plants: High-speed soft robotic actuators. Adv. Sci. **7**, 1903391 (2020). 10.1002/advs.201903391 PMC7055565 32154089

[9] D. Yang , Buckling pneumatic linear actuators inspired by muscle. Adv. Mater. Technol. **1**, 1600055 (2016).

[10] D. Yang , Buckling of elastomeric beams enables actuation of soft machines. Adv. Mater. **27**, 6323–6327 (2015). 26389733 10.1002/adma.201503188

[11] J. Shi, H. Mofatteh, A. Mirabolghasemi, G. Desharnais, A. Akbarzadeh, Programmable multistable perforated shellular. Adv. Mater. **33**, 2102423 (2021). 10.1002/adma.202102423 34467581

[12] S. Shan , Multistable architected materials for trapping elastic strain energy. Adv. Mater. **27**, 4296–4301 (2015). 26088462 10.1002/adma.201501708

[13] D. Restrepo, N. D. Mankame, P. D. Zavattieri, Phase transforming cellular materials. Extrem. Mech. Lett. **4**, 52–60 (2015). 10.1038/s41598-019-48581-8 PMC6715794 31467381

[14] L. Jin, A. E. Forte, B. Deng, A. Rafsanjani, K. Bertoldi, Kirigami-inspired inflatables with programmable shapes. Adv. Mater. **32**, 2001863 (2020). 10.1002/adma.202001863 32627259

[15] S. Mhatre , Deployable structures based on buckling of curved beams upon a rotational input. Adv. Funct. Mater. **31**, 2101144 (2021).

[16] V. S. C. Chillara, A. K. Ramanathan, M. J. Dapino, Self-sensing piezoelectric bistable laminates for morphing structures. Smart Mater. Struct. **29**, 085008 (2020).

[17] H. Le Ferrand, A. R. Studart, A. F. Arrieta, Filtered mechanosensing using snapping composites with embedded mechano-electrical transduction. ACS Nano **13**, 4752–4760 (2019). 30925044 10.1021/acsnano.9b01095

[18] T. G. Thuruthel, S. Haider Abidi, M. Cianchetti, C. Laschi, E. Falotico, A bistable soft gripper with mechanically embedded sensing and actuation for fast grasping in 2020 29th IEEE International Conference on Robot and Human Interactive Communication (RO-MAN), (IEEE, 2020), pp. 1049–1054.

[19] O. Peretz, A. K. Mishra, R. F. Shepherd, A. D. Gat, Underactuated fluidic control of a continuous multistable membrane. Proc. Natl. Acad. Sci. U.S.A. **117**, 5217–5221 (2020). 32094198 10.1073/pnas.1919738117 PMC7071869

[20] Y. Jiang, L. M. Korpas, J. R. Raney, Bifurcation-based embodied logic and autonomous actuation. Nat. Commun. **10**, 128 (2019). 30631058 10.1038/s41467-018-08055-3 PMC6328580

[21] P. Rothemund , A soft, bistable valve for autonomous control of soft actuators. Sci. Robot. **3**, eaar7986 (2018). 33141749 10.1126/scirobotics.aar7986

[22] D. J. Preston , Digital logic for soft devices. Proc. Natl. Acad. Sci. U.S.A. **116**, 7750–7759 (2019). 30923120 10.1073/pnas.1820672116 PMC6475414

[23] T. Mei, Z. Meng, K. Zhao, C. Q. Chen, A mechanical metamaterial with reprogrammable logical functions. Nat. Commun. **12**, 7234 (2021). 34903754 10.1038/s41467-021-27608-7 PMC8668933

[24] D. Yang , Phase-transforming and switchable metamaterials. Extrem. Mech. Lett. **6**, 1–9 (2016).

[25] D. Goswami, S. Liu, A. Pal, L. G. Silva, R. V. Martinez, 3D-architected soft machines with topologically encoded motion. Adv. Funct. Mater. **29**, 1808713 (2019).

[26] B. Haghpanah, L. Salari-Sharif, P. Pourrajab, J. Hopkins, L. Valdevit, Multistable shape-reconfigurable architected materials. Adv. Mater. **28**, 7915–7920 (2016). 27384125 10.1002/adma.201601650

[27] J. R. Raney , Stable propagation of mechanical signals in soft media using stored elastic energy. Proc. Natl. Acad. Sci. U.S.A. **113**, 9722–9727 (2016). 27519797 10.1073/pnas.1604838113 PMC5024640

[28] H. Yasuda, L. M. Korpas, J. R. Raney, Transition waves and formation of domain walls in multistable mechanical metamaterials. Phys. Rev. Appl. **13**, 054067 (2020).

[29] N. Vasios, B. Deng, B. Gorissen, K. Bertoldi, Universally bistable shells with nonzero Gaussian curvature for two-way transition waves. Nat. Commun. **12**, 695 (2021). 33514707 10.1038/s41467-020-20698-9 PMC7846611

[30] L. Jin , Guided transition waves in multistable mechanical metamaterials. Proc. Natl. Acad. Sci. U.S.A. **117**, 2319–2325 (2020). 31969454 10.1073/pnas.1913228117 PMC7007517

[31] N. Nadkarni, A. F. Arrieta, C. Chong, D. M. Kochmann, C. Daraio, Unidirectional transition waves in bistable lattices. Phys. Rev. Lett. **116**, 244501 (2016). 27367390 10.1103/PhysRevLett.116.244501

[32] Y. Song , Additively manufacturable micro-mechanical logic gates. Nat. Commun. **10**, 882 (2019). 30787283 10.1038/s41467-019-08678-0 PMC6382908

[33] H. Fang, K. W. Wang, S. Li, Asymmetric energy barrier and mechanical diode effect from folding multi-stable stacked-origami. Extrem. Mech. Lett. **17**, 7–15 (2017).

[34] A. Zareei, B. Deng, K. Bertoldi, Harnessing transition waves to realize deployable structures. Proc. Natl. Acad. Sci. U.S.A. **117**, 4015–4020 (2020). 32041876 10.1073/pnas.1917887117 PMC7049108

[35] B. Deng, J. R. Raney, K. Bertoldi, V. Tournat, Nonlinear waves in flexible mechanical metamaterials. J. Appl. Phys. **130**, 040901 (2021).

[36] B. Deng, J. R. Raney, V. Tournat, K. Bertoldi, Elastic vector solitons in soft architected materials. Phys. Rev. Lett. **118**, 204102 (2017). 28581775 10.1103/PhysRevLett.118.204102

[37] B. Deng, P. Wang, Q. He, V. Tournat, K. Bertoldi, Metamaterials with amplitude gaps for elastic solitons. Nat. Commun. **9**, 3410 (2018). 30143618 10.1038/s41467-018-05908-9 PMC6109112

[38] H. Yasuda , Origami-based impact mitigation via rarefaction solitary wave creation. Sci. Adv. **5**, eaau2835 (2019). 31139744 10.1126/sciadv.aau2835 PMC6534386

[39] F. Fraternali, G. Carpentieri, A. Amendola, R. E. Skelton, V. F. Nesterenko, Multiscale tunability of solitary wave dynamics in tensegrity metamaterials. Appl. Phys. Lett. **105**, 201903 (2014).

[40] M. Hwang, A. F. Arrieta, Input-independent energy harvesting in bistable lattices from transition waves. Sci. Rep. **8**, 3630 (2018). 29483610 10.1038/s41598-018-22003-7 PMC5827759

[41] Y. Zhang, M. Velay-Lizancos, D. Restrepo, N. D. Mankame, P. D. Zavattieri, Architected material analogs for shape memory alloys. Matter **4**, 1990–2012 (2021).

[42] H. Niknam, A. Akbarzadeh, D. Therriault, S. Bodkhe, Tunable thermally bistable multi-material structure. Appl. Mater. Today **28**, 101529 (2022).

[43] G. Librandi, E. Tubaldi, K. Bertoldi, Programming nonreciprocity and reversibility in multistable mechanical metamaterials. Nat. Commun. **12**, 3454 (2021). 34103522 10.1038/s41467-021-23690-z PMC8187725

[44] H. Yasuda, T. Tachi, M. Lee, J. Yang, Origami-based tunable truss structures for non-volatile mechanical memory operation. Nat. Commun. **8**, 962 (2017). 29042544 10.1038/s41467-017-00670-w PMC5714951

[45] I. Mahboob, H. Yamaguchi, Bit storage and bit flip operations in an electromechanical oscillator. Nat. Nanotechnol. **3**, 369–369 (2008). 10.1038/nnano.2008.84 18654523

[46] O. R. Bilal, A. Foehr, C. Daraio, Bistable metamaterial for switching and cascading elastic vibrations. Proc. Natl. Acad. Sci. U.S.A. **114**, 4603–4606 (2017). 28416663 10.1073/pnas.1618314114 PMC5422829

[47] A. Ion, L. Wall, R. Kovacs, P. Baudisch, Digital mechanical metamaterials in Proceedings of the 2017 CHI Conference on Human Factors in Computing Systems (ACM, 2017), pp. 977–988.

[48] G. Z. Lum , Shape-programmable magnetic soft matter. Proc. Natl. Acad. Sci. U.S.A. **113**, E6007–E6015 (2016). 27671658 10.1073/pnas.1608193113 PMC5068264

[49] W. Hu, G. Z. Lum, M. Mastrangeli, M. Sitti, Small-scale soft-bodied robot with multimodal locomotion. Nature **554**, 81–85 (2018). 29364873 10.1038/nature25443

[50] Y. Alapan, A. C. Karacakol, S. N. Guzelhan, I. Isik, M. Sitti, Reprogrammable shape morphing of magnetic soft machines. Sci. Adv. **6**, eabc6414 (2020). 32948594 10.1126/sciadv.abc6414 PMC7500935

[51] J. Zhang , Voxelated three-dimensional miniature magnetic soft machines via multimaterial heterogeneous assembly. Sci. Robot. **6**, eabf0112 (2021). 34043568 10.1126/scirobotics.abf0112 PMC7612277

[52] B. Moran , Field responsive mechanical metamaterials. Sci. Adv. **4**, eaau6419 (2018). 30539147 10.1126/sciadv.aau6419 PMC6286172

[53] Z. Liu , Creating three-dimensional magnetic functional microdevices via molding-integrated direct laser writing. Nat. Commun. **13**, 2016 (2022). 35440590 10.1038/s41467-022-29645-2 PMC9019016

[54] T. Chen, M. Pauly, P. M. Reis, A reprogrammable mechanical metamaterial with stable memory. Nature **589**, 386–390 (2021). 33473228 10.1038/s41586-020-03123-5

[55] A. Abbasi, T. G. Sano, D. Yan, P. M. Reis, Snap buckling of bistable beams under combined mechanical and magnetic loading. Philos. Trans. R. Soc. A Math. Phys. Eng. Sci. **381** (2023), 10.1098/rsta.2022.0029. 36774950

[56] M. Sitti, Physical intelligence as a new paradigm. Extrem. Mech. Lett. **46**, 101340 (2021). PMC7612657 35475112

[57] H. W. Sung, C. Rudowicz, Physics behind the magnetic hysteresis loop—a survey of misconceptions in magnetism literature. J. Magn. Magn. Mater. **260**, 250–260 (2003).

