# Supplementary material for: Programmable mechanical devices through magnetically tunable bistable elements
Source: Proc Natl Acad Sci U S A. 2023 Apr 3;120(15):e2212489120. doi: 10.1073/pnas.2212489120 (PMC10104571; doi:10.1073/pnas.2212489120)
Supplement: Supplementary file 1 — Appendix 01 (PDF) [file pnas.2212489120.sapp.pdf]

Supplementary Information for

Programmable mechanical devices through magnetically tunable bistable elements

Aniket Pal and Metin Sitti

Corresponding Author: Metin Sitti
Email: sitti@is.mpg.de

This PDF file includes:

Supplementary text
Figures S1 to S8
Captions for Movies S1 to S7

Other supplementary materials for this manuscript include the following:

Movies S1 to S7

Supporting Information Text

1. Materials

Neodymium-iron-boron (NdFeB) magnetic microparticles with an average diameter of 5 μm was used (MQP-15-7, Magnequench International, LLC) as the active magnetic material in all our experiments. Polydimethylsiloxane (PDMS) (Sylgard 184) and Ecloflex 00-30 (Smooth-On, Inc.) were used as elastomeric materials. Silicone Thinner™ (Smooth-On, Inc.) was added to the uncured silicone composites (1:10 w/w) to decrease the viscosity of uncured elastomers. A silicone adhesive (Sil-Poxy™, Smooth-On, Inc.) was used to join different elastomeric components. Universal™ Mold Release (Smooth-On, Inc.) was used as a release agent to ensure easy removal of elastomers from 3D printed molds. Nickel-plated, NdFeB block permanent magnets of size 5x5x1 mm^3 and 10x5x1 mm^3 (Q-CDM48-N and Q-10-05-01-STIC, supermagnete) were used to create the desired external magnetic fields. The residual magnetisms of the two magnet types were between 1.37-1.42 T and 1.17-1.21 T respectively.

2. Fabrication of magneto-elastomeric composites

All magneto-elastomeric composites were fabricated by mixing and degassing magnetic microparticles in uncured elastomers in a planetary mixer and deaerator (KK-250SE, Kurabo Mazerustar) for 90 s. We used two elastomers, PDMS and Ecoflex-0030, and 10% by weight of silicone thinner was added in both cases to reduce the viscosity and ensure uniform distribution of the magnetic microparticles. The magnetic microparticles were added in a 2:1 ratio (by weight) to the elastomers (excluding the thinner). A mold with uniform height was made by attaching multiple layers of a heat-resistant, polyester backing tape with known thickness as the four sides, on top of a poly(methyl methacrylate) (PMMA) substrate. The uncured elastomer mixture was poured in the mold and doctor-bladed to ensure uniform thickness. The PDMS based elastomeric composite required four hours, while the Ecoflex-0030 based elastomeric composite required two hours to completely cure in a 90 °C oven. The cured sheets were not tacky to the touch and had a smooth finish.

3. Fabrication of bistable beams

We laser cut the cured sheet with a solid-state laser (355 nm, ProtoLaser U3, LPKF Laser & Electronics) to get beams in the required dimensions. All beams in our experiments and simulations had an area dimension of 14x4 mm^2, while the thickness was varied. 4 mm of the length of the beams (2 mm on each side) was adhered to its bracket/frame, leaving a 10 mm free length. An exception were the beams with magnetization direction normal to their length,

they had an area dimension of 14x14 mm^2, which eventually led to a 10x14 mm^2 free beam. Having the width bigger than the length ensured that the beam would bend along its length, instead of twisting about its length. The beams were attached to their brackets/frames using a silicone adhesive. The softness of the silicone adhesive allowed some (finite) stretching, which is why the experiments would often record lower forces at high strains compared to simulations (Fig. 3C, S2). The frames themselves were 3D printed (J835, Stratasys, Ltd.**)** with a rigid material (Vero PureWhite™, RGD837).

4. Finite element analysis (FEA)

FEA was performed to model the mechanical responses of magneto-elastomeric beams in different uniform magnetic fields. All FEA simulations were performed with a commercial finite element solver (Abaqus/Standard, version 2020, Simulia, Dassault Systèmes) using 2D shell elements of type S4. Taking advantage of the symmetry of the beams, one half of the beams were simulated to increase computational efficiency. The fixed end was fixed using "encastre" boundary conditions, while the other end utilized symmetry boundary conditions. Each full beam was deformed by applying a vertical displacement to the center and the generated reaction force was recorded. The force-displacement data was used to calculate the energy as described: First, we fit the numerically obtained force-displacement curve with a polynomial of the degree 10. We then integrated the polynomial between its roots (real roots, which were within the displacement limits) to obtain the E_{in} and E_{out} values.

The snapshots of FE results show the full beam by mirroring the simulated half. The t/L ratios used in out experiments are compatible with 2D elements and shell theory. Dynamic/Implicit analyses were performed to capture the instabilities and snap-through behaviors of the magneto-elastomeric beams. The kinetic energy of the simulations was monitored and a small damping factor was introduced to ensure quasi-static conditions. The magnetic torques on the beams were calculated with a custom user subroutine (UAMP), which was used to update the magnitude and direction of 3D torque values at each node, based on their 3D orientations at each increment. Specifically, a *History Output* was set up for each of the three rotational degrees of freedom for each node, with an output frequency of every iteration. This *History Output* data was read as a *Sensor* by the custom UAMP (user-defined amplitude) subroutine to determine the orientation of each node at every iteration. The orientation was used to calculate the magnetic torque being acted on each node, which was then communicated as an amplitude for a *Moment* (for each axis) for every node. After the next iteration, along with all other model

parameters, the orientation of the nodes was also updated and communicated to the UAMP subroutine to calculate the torque for the next iteration. A high number of iterations were used to mimic real life responses and prevent oscillation of the beam. The external magnetic field was considered to be uniform throughout the beam deformation region, thus gradient based magnetic forces were not relevant.

5. Mechanical characterization of magneto-elastomeric composites

Tensile tests of dogbone-shaped samples of the magneto-elastomeric materials were performed according to ASTM D412-C specifications to determine the elastic modulus of the materials. A universal testing machine (Instron 5942), with 10 N and 50 N load cells, was used to perform the experiments at a crosshead loading rate of 5 mm min^{-1} (sampling rate 50 Hz). The materials were found to be linearly elastic with an elastic modulus of 44 kPa and 4.9 MPa for the Ecoflex and PDMS based magneto-elastomeric composites, respectively (Fig. S5A, B).

The stress-relaxation experiments were also performed in the same universal testing machine by applying a fast, instantaneous displacement (2 mm @ 60 mm min^{-1}), causing a strain of 20%, and the force was measured with a 10 N load cell (sampling rate 50 Hz) for 300 s. We then fitted the Maxwell form of the standard linear solid (SLS) model of linear viscoelasticity to our experimental data, where the instantaneous force (F(t)) can be represented as $F(t) = F_0 + F_1 e^{-t/\tau}$, where F_0 and F_1 are the time independent and time dependent components of the total force respectively and τ is the time constant (Fig. S5C, D). Our SLS model had a high correlation with the experimental data ($r = 0.975$) and showed a very low F_1/F_0 ratio (0.066), indicating that the material demonstrates primarily pure elastic behavior.

6. Uniform magnetic field from permanent magnets

We used Nickel-plated, NdFeB block permanent magnets to create the desired external magnetic fields. A bistable beam, with two magnets on either side of it, experienced primarily uniform magnetic fields with minimal gradients (Fig. S7). The magnetic field was in our region of interest was derived numerically (through FE simulations with COMSOL Multiphysics®, version 6.0) as well as measured experimentally with a Gaussmeter (HGM09s, MAGSYS magnet systeme GmbH, resolution 1 mT), showing close agreement between the measured and simulated magnetic fields. The sampling point for both numerical and experimental results were distributed uniformly throughout the active workspace. For the experimental

measurements the probe could not physically reach the bottom of the workspace, hence the measurements at the top were weighted double to obtain unbiased statistics. To create magnetic fields applicable to multiple bistable beams, the permanent magnet configuration previously described was linearly arrayed, creating uniform magnetic fields for each element.

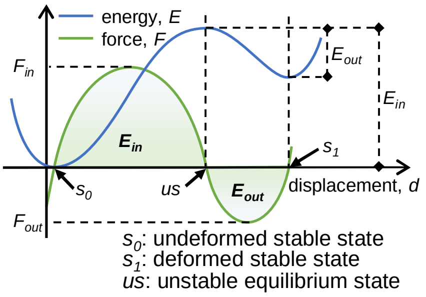

Fig. S1. Representative force- and energy-displacement curves of a bistable element. The two stable states are denoted by s_0 and s_1. F_{in} and F_{out} are the maximum positive and negative force respectively. E_{in} and E_{out} are the energy absorbed and released by the system. F_{out} and E_{out} are zero if the system is not bistable.

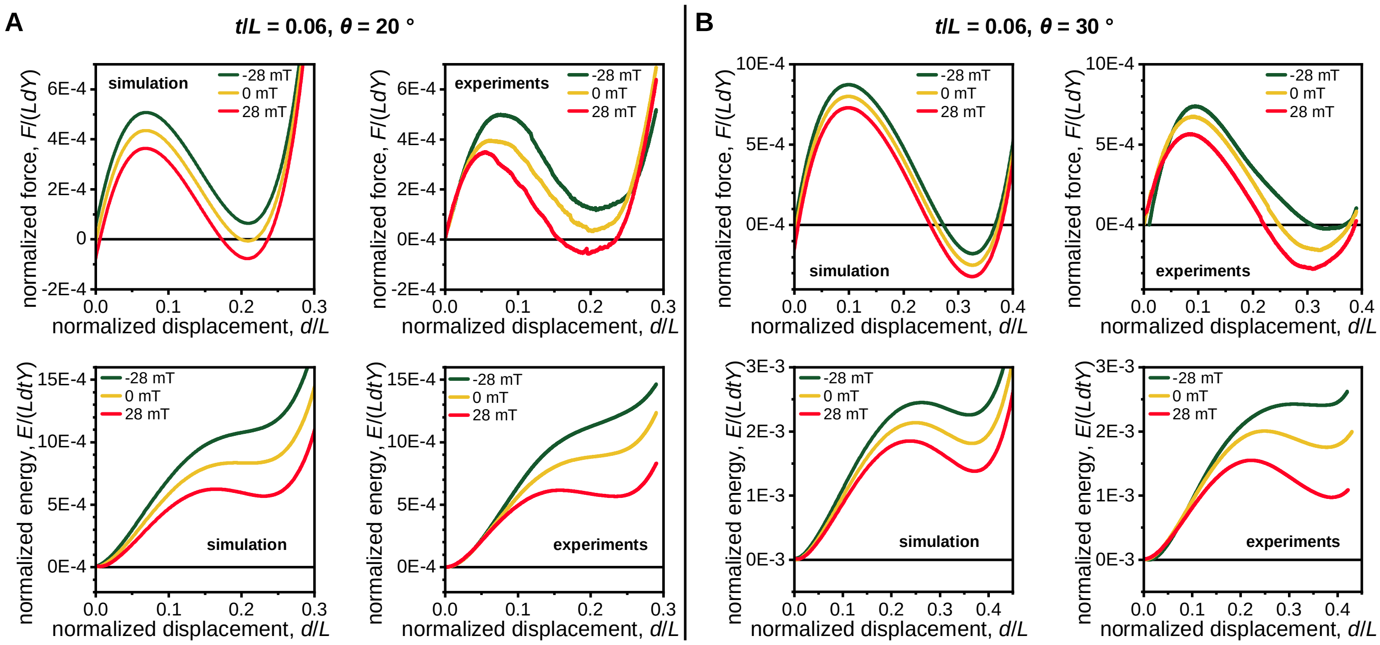

Fig. S2. Magnetically tuned responses of magneto-elastomeric beams magnetized symmetrically along its axis and with different geometries. (A, B) Normalized force and energy profiles, from experiments and FE simulations, of magneto-elastomeric beams with θ = 20°, t/L = 0.06 (A), and θ = 30°, t/L = 0.06 (B) under varying magnetic fields.

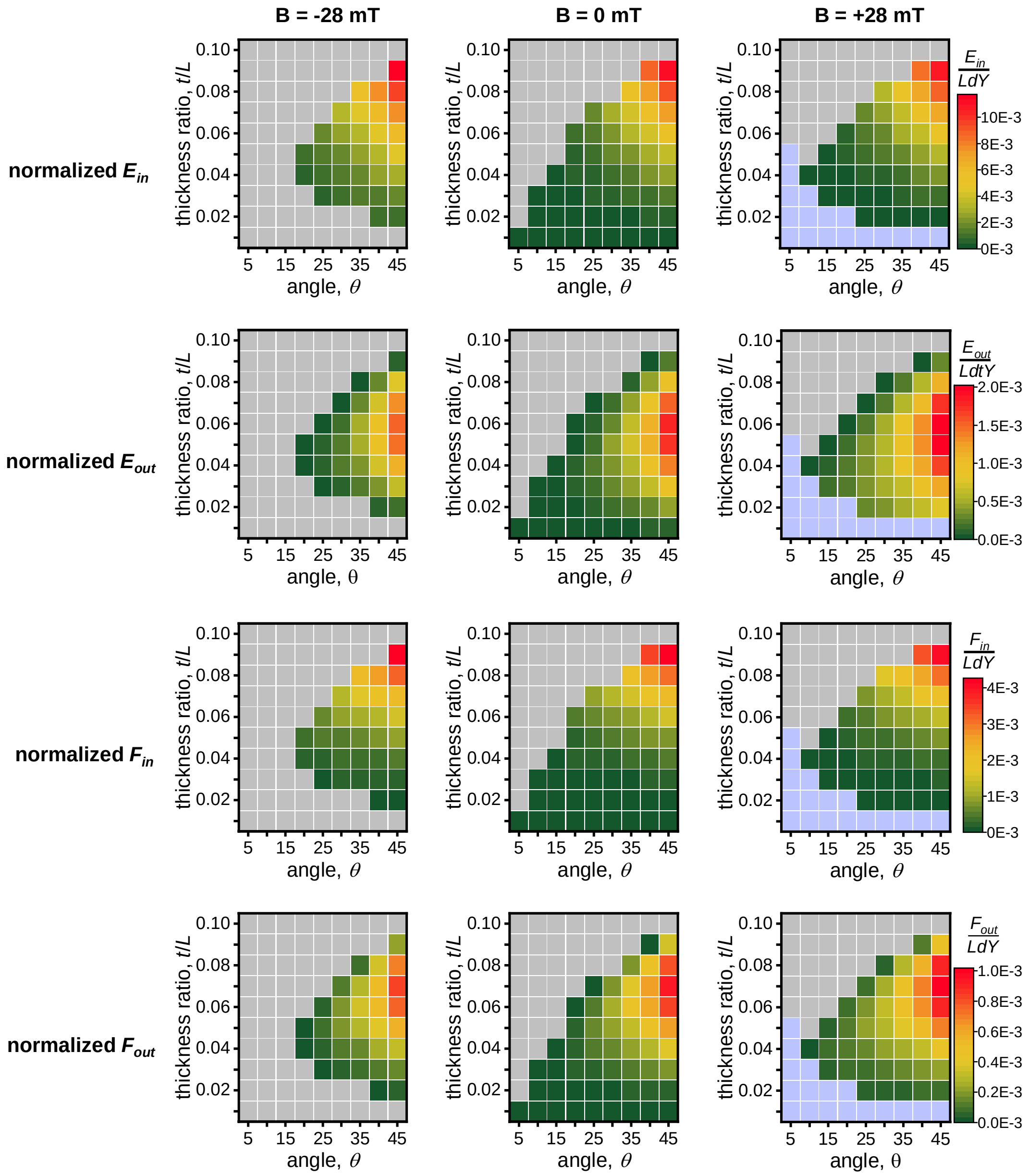

Fig. S3. Effect of *θ* and *t*/*L* on the normalized maximum output force and normalized energy released by the system while transitioning to the second stable state (B_z = 0, -28, and +28 mT). The gray regions indicate combinations where bistability was not achieved. The blue regions indicate where the s_1 became the only stable state.

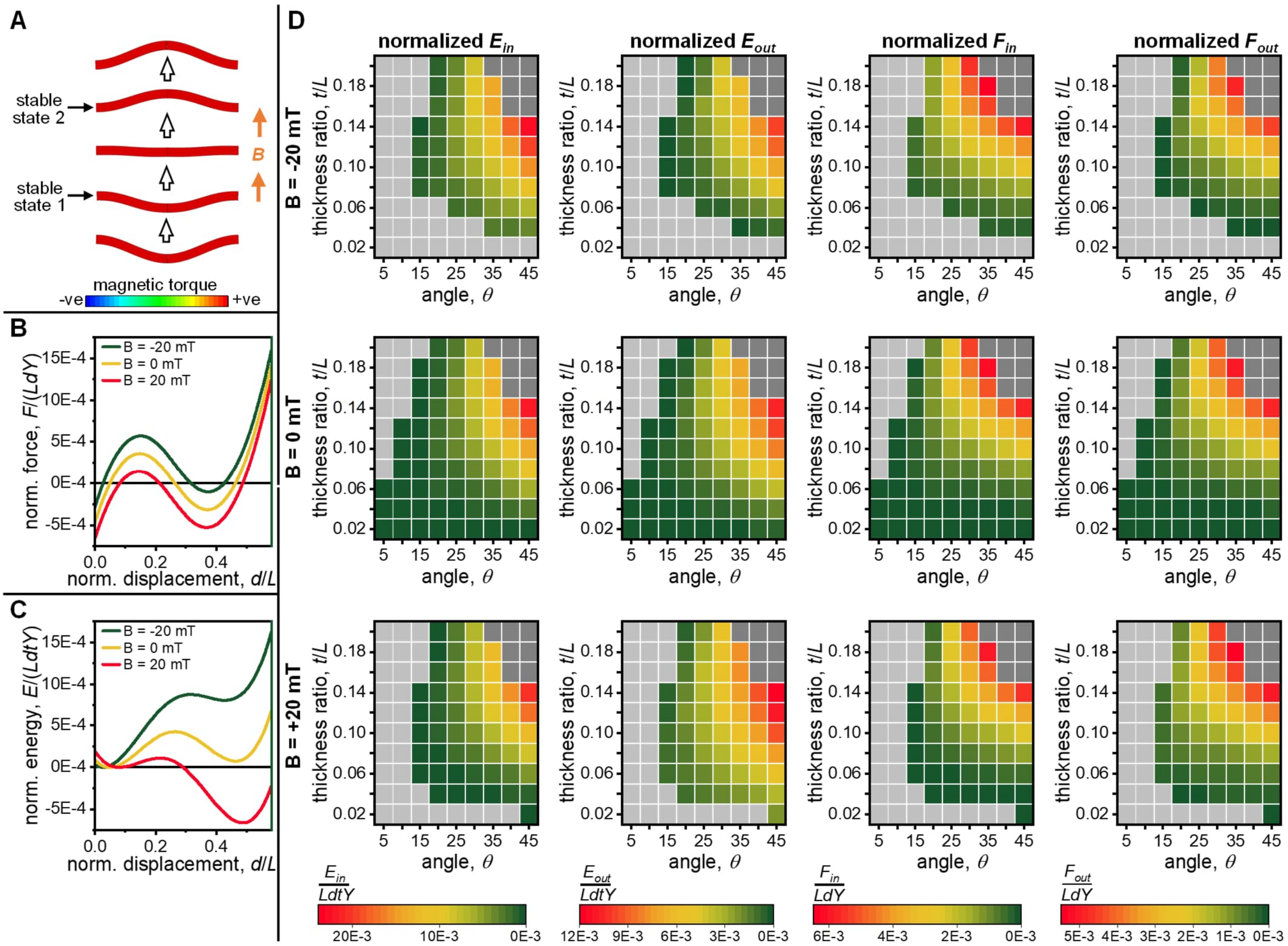

Fig. S4. Magneto-elastomeric bistable beams with straight fixed ends. (A) Mirrored FE simulations results showing the evolution of the torques generated along the beam (under to a magnetic field of B_z = 20mT) as it is displaced vertically. (B, C) Normalized force (B) and energy (C) profiles of a magneto-elastomeric beam (initial angle, θ = 15° and thickness-to-length, t/L = 0.06) under varying magnetic fields. (D) Parametric study showing the values of E_{in}, E_{out}, F_{in}, and F_{out} due to varying θ and t/L, under different magnetic fields (B_z = 0, -20, and +20 mT).

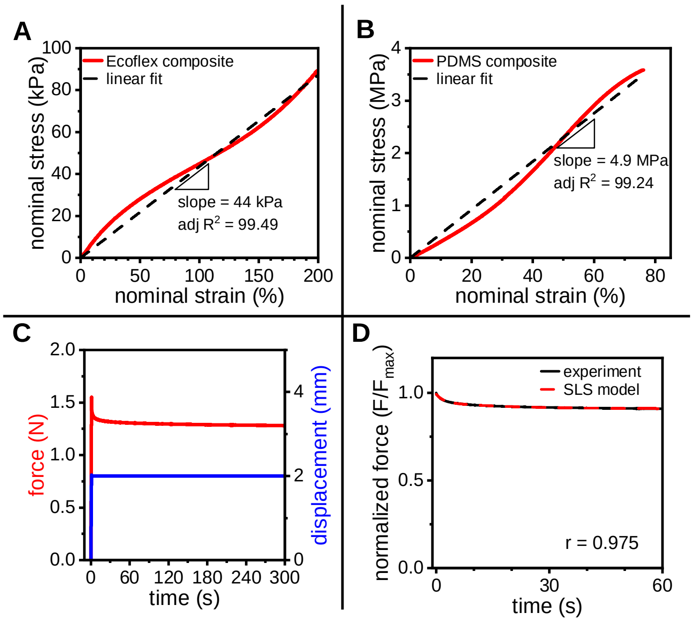

Fig. S5. Mechanical characterization of the magneto-elastomeric composites. (A, B) Representative stress-strain curves of dogbone-shaped sample of Ecoflex (A) and PDMS (B) based magneto-elastic composite obtained from a uniaxial tensile test. The slope of the linear fit indicates the elastic modulus of the materials (44 kPa and 4.9 MPa for the Ecoflex and PDMS based magneto-elastomeric composites respectively. (C) Stress relaxation of the PDMS composite where a constant displacement is applied to the sample and the force is measure for 300 s. (D) Fitted Maxwell form of the standard linear solid (SLS) model of linear viscoelasticity (correlation coefficient r = 0.975).

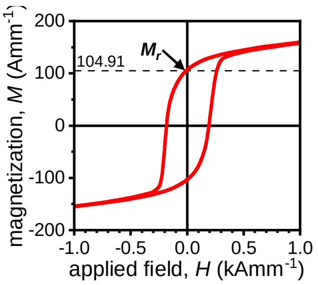

Fig. S6. Magnetic characterization of the magneto-elastomeric composites. Representative magnetic hysteresis curve under cyclical external magnetic field. The intersection of the magnetization curve with the zero applied field line indicated the remnant magnetization, M_r.

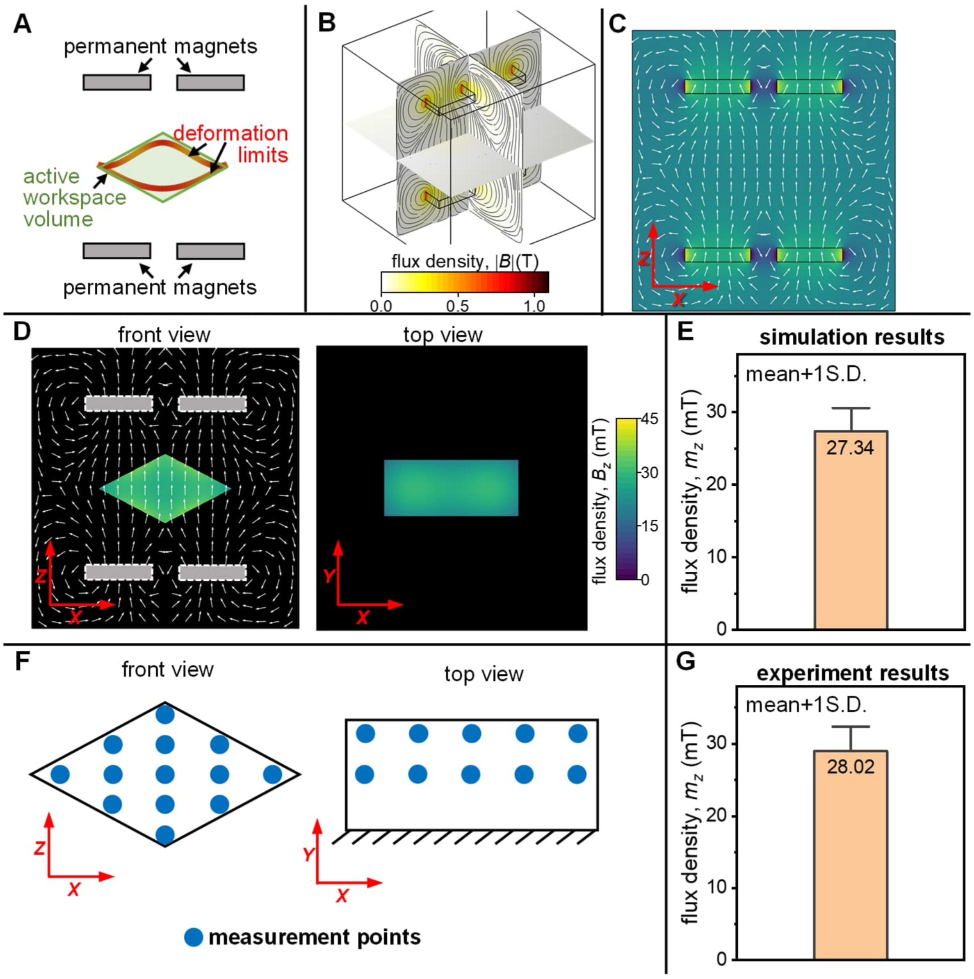

Fig. S7. Uniform magnetic field generated from permanent magnets. (A) Experimental setup showing the configuration of the permanent magnets and the active workspace volume, defined by the limits of the beam deformation. (B) 3D simulation results showing the magnetic flux density distribution. (C) 2D simulation results showing the in-plane flux density distribution (***B***). The arrows indicate the direction of the flux, the heatmap indicates the magnitude of only the *Z* component of the flux (B_z). (D) Front and top view of the magnetic flux density inside the active workspace volume. (E) Mean (27.34 mT) and standard deviation (3.28 mT) of B_z inside the active workspace volume calculated by sampling at 3600 uniformly spaced points. (F) Front and top view of the sample points for experimental measurements inside the active workspace volume. (E) Mean (28.02 mT) and standard deviation (4.06 mT) of B_z inside the active workspace volume measured at 26 points.

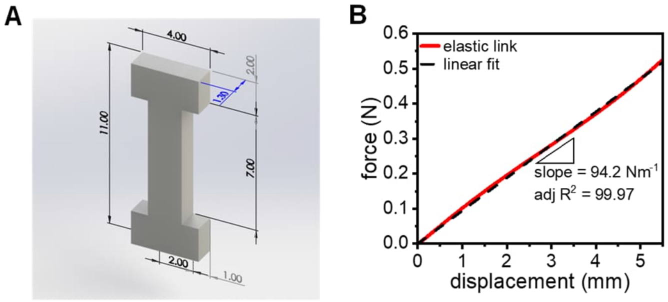

Fig. S8. Details of the elastic link. (A) Detailed sketch with dimension of the elastic link. (B) tensile test results of the elastic link demonstrating a linear response with a spring constant of 94 N m^{-1}.

Supplementary Movies

Movie S1: Propagation of a transition wave in a lattice of bistable elements

Movie S2: Transistor like control of transition wave propagation

Movie S3: Magnetic control of velocity of transition waves

Movie S4: magnetic control of the direction of propagation of transition waves

Movie S5: Magnetic control of the functionality of a binary logical element: **OR** gate

Movie S6: Magnetic control of the functionality of a binary logical element: **AND** gate

Movie S7: Introducing bistability in an inherently monostable beam
